# Optimising (Al,Ga) (As,Bi) Quantum Well Laser Structures for Reflectance Mode Pulse Oximetry

**DOI:** 10.3390/mi16050506

**Published:** 2025-04-26

**Authors:** Aivaras Špokas, Andrea Zelioli, Andrius Bičiūnas, Bronislovas Čechavičius, Justinas Glemža, Sandra Pralgauskaitė, Mindaugas Kamarauskas, Virginijus Bukauskas, Janis Spigulis, Yi-Jen Chiu, Jonas Matukas, Renata Butkutė

**Affiliations:** 1 Center for Physical Sciences and Technology, 10257 Vilnius, Lithuania; andrea.zelioli@ftmc.lt (A.Z.); andrius.biciunas@ftmc.lt (A.B.); bronislovas.cechavicius@ftmc.lt (B.Č.); mindaugas.kamarauskas@ftmc.lt (M.K.); virginijus.bukauskas@ftmc.lt (V.B.); 2 Institute of Photonics and Nanotechnology, Faculty of Physics, Vilnius University, 10257 Vilnius, Lithuania; 3 Institute of Applied Electrodynamics and Telecommunications, Faculty of Physics, Vilnius University, 10257 Vilnius, Lithuania; justinas.glemza@ff.vu.lt (J.G.); sandra.pralgauskaite@ff.vu.lt (S.P.); jonas.matukas@ff.vu.lt (J.M.); 4 Biophotonics Laboratory, Institute of Atomic Physics and Spectroscopy, University of Latvia, 1004 Riga, Latvia; janis.spigulis@lu.lv; 5 Institute of Electro-Optical Engineering, Department of Photonics, National Sun Yat-Sen University, Kaohsiung 804, Taiwan; yjchiu@mail.nsysu.edu.tw

**Keywords:** GaAsBi, QW with parabolic graded barriers, laser diode

## Abstract

We explore quantum well laser diodes for applications in pulse oximetry based on two material systems, namely, classical AlGaAs and a rather exotic GaAsBi, with lasing at around 800 nm and 1100 nm, respectively. These spectral regions and material families were selected due to their closely matched effective penetration depths into soft tissue. An improved design of the band structure of device active areas was tested on both material systems, yielding enhancement of the two main parameters, namely, output power and threshold current. A maximum emission power of the AlGaAs laser diode was registered at 4.9 mW (I = 60 mA, λ = 801 nm). For the GaAsBi-based devices, the target emission of 1106 nm was measured in pulsed mode with a peak output power of 9.4 mW (I = 3 A). The most optimized structure was based on three GaAsBi quantum wells surrounded by parabolically graded AlGaAs barriers. This structure was capable of 130 mW peak power (I = 2 A, λ = 1025 nm) along with a more than tenfold decrease in threshold current to 250 mA compared to a classical rectangular quantum well active region.

## 1. Introduction

The ever-growing demand for monitoring systems of human vitals drives the improvement of the components of integrated sensor systems. One area of possible improvement is pulse oximetry. Traditionally, 660 nm red and 940 nm near-infrared (NIR) emitters have been used for oximetry applications [1]. While such technology is capable of achieving satisfactory results in transmission measurements (e.g., finger clips), the accuracy of measurements in reflectance mode is hindered by the difference in penetration depth of these wavelengths. It has been demonstrated that an improvement could be achieved by using two NIR laser diodes (LD) with similar emission wavelengths, resulting in a similar optical path length. Nonetheless, using nearby emission lines results in technological challenges [2]. This underscores the need for further research and development in the field. Another solution can involve using two NIR wavelengths in the vicinity of 800 nm and 1100 nm, as these are far apart and have a similar penetration depth into soft tissue. AlGaAs quantum well (QW) laser diodes are the obvious candidate for the 800 nm emission line, while the choice of emitters operating at 1100 nm is more complicated. The classical material for this wavelength region is InGaAs; however, structures with over 20% of In are necessary for emission at 1100 nm, and these are prone to relaxation and formation of a dislocation grid due to the large lattice mismatch with GaAs substrates. GaAsBi QW-based structures can be used to overcome this issue, and also have many attractive properties for applications in laser diode active media. First of all, the introduction of Bi into the lattice of GaAs induces a substantial band gap reduction of up to 88 meV/%Bi [3]. This means that a small amount of host As atoms have to be replaced in order to achieve a large redshift without inducing strain in the structure, which could result in dislocations that limit the applicability in devices. Additionally, the increase in Bi content also increases the spin–orbit split-off (ΔSO) energy due to major influence on the valance band. At a Bi content of 10.5% in GaAsBi, the ΔSO becomes larger than the band gap energy; in this way, a major Auger non-radiative recombination path can be suppressed [4]. Finally, bismides possess temperature-insensitive emission energy paired with stable room-temperature operation of GaAsBi-based devices, making cooling less crucial [5,6]. The latter properties are significant for device applications that are supposed to work in various environmental conditions and maintain the same accuracy levels, making GaAsBi the perfect candidate for application-focused 1100 nm emitters. The ability of GaAsBi to achieve lasing at longer wavelengths has been demonstrated by Wu et al., who achieved 1142 nm emission with a GaAsBi QW laser diode [7]. Even though GaAsBi-based laser diodes have been successfully produced by many groups [7,8,9,10,11,12], their wider application is limited by the defective crystalline structure of the alloy, which negatively affects the reproducibility, threshold current (*I*_th_), and maximum power output of the devices. Some of these defects consist of Bi_Ga_ antisites [13]. Bi clustering has also been shown to be energetically favored in Bi atom ordering [14], and CuPt_B_-type ordering has been noticed in bismides, resulting in optical anisotropy of the structure. Furthermore, Gelczuk et al. reported electron trap levels in n-type GaAsBi that are crucial for device performance [15], where two out of the six trap states are related to Bi (specifically a complex defect containing a Bi_Ga_ antisite) [13]. The other four trap levels are associated with low-temperature GaAs, which have been shown to be a prominent current leakage channel in GaAsBi LDs [16]. The major cause of these defects is the complicated growth of the alloy, which is usually conducted at a stoichiometric As-to-Ga flux ratio and a low growth temperature that also doubles as a limiting factor for Bi incorporation. This means that high Bi content is achieved at extremely low growth levels, resulting in large defect density [17,18,19]. Several strategies have been employed to improve the performance of GaAsBi lasers. Marko et al. reported a decreased threshold current when introducing Al into the barriers [10]. Our earlier work focused on enhancing the emission characteristics of quantum structures based both on pure AlGaAs and on a GaAsBi alloy containing high Bi by designing parabolically graded barriers [20].

In this work, we explore the opportunity of application of two laser diode families, namely, AlGaAs and GaAsBi, in pulse oximetry, with the aim of reaching the target wavelengths of about 800 nm and 1100 nm. GaAsBi QW–based laser diodes were optimized for emission at around 1100 nm, while AlGaAs QW structures based on the classical material approach were produced for emission in the vicinity of 800 nm, providing a reference for laser diode performance. Structures with both of the investigated compounds were produced with parabolically graded barriers to increase the maximum output power and decrease the *I*_th_.

## 2. Structure Modeling and Growth

Two material systems were utilized to produce the presented laser structures. The AlGaAs alloy was selected for the shorter target wavelength due to its straightforward and well-established growth. On the other hand, a quite exotic material system, GaAsBi, was employed for the longer wavelength spectral region. Additionally, two different designs of the active area band structure, rectangular and parabolically graded barriers (PGBs), were developed for both material systems to clarify the beneficial impact of the PGBs on the device performance parameters. The laser structures in this work are named according to their structure design, with the first letter representing the geometry (R—standard rectangular; P—parabolic) and the second two letters representing the material system (Bi—GaAsBi; Al—AlGaAs).

The structures were simulated using nextnano++ software (Nextnano GmbH, München, Germany), allowing for comprehensive full-band quantum mechanical modeling. A one-dimensional (1D) simulation was conducted along the growth direction (z-axis). The electronic band structure was calculated employing the 8 × 8 k·p method within the effective mass approximation framework [21]. Only the Γ-point was considered the conduction band minimum, and the two valence band maxima, corresponding to the heavy hole (hh) and light hole (lh) states, were both included in the calculations. Additionally, excitonic effects and temperature-induced variations of the band gap were incorporated into the simulations. Due to the matched lattice constants of AlGaAs and GaAs, strain-related effects were omitted from the model. The simulation parameters for GaAsBi, band gap, ΔSO, elastic constant, lattice constant, and band offset were obtained from the literature [22,23,24,25].

**Figure 1:**
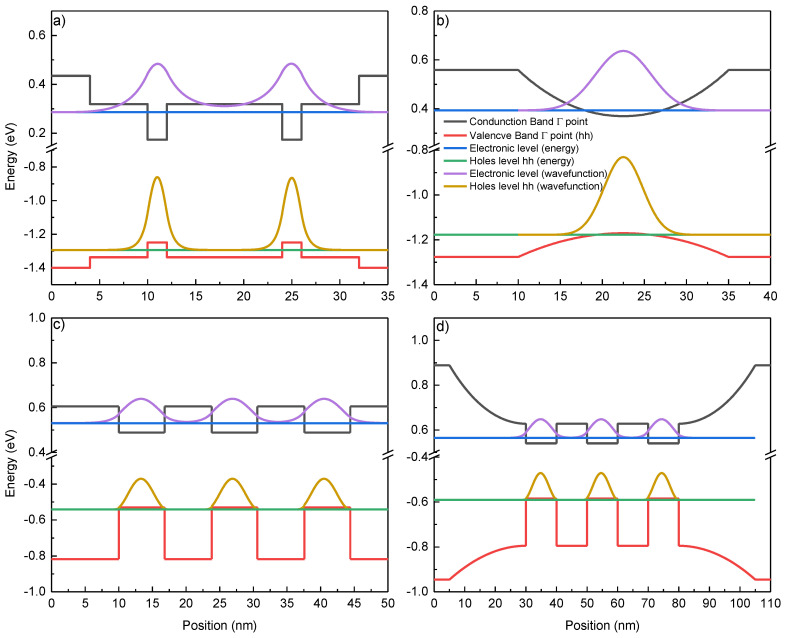
nextnano++ models of the intrinsic regions of the laser diode: (**a**) RAl, (**b**) PAl, (**c**) RBi, and (**d**) PBi. The legend is for all four graphs.

Figure 1 illustrates the calculated band edges of four active areas of the studied laser diodes. Figure 1a presents the gain region of structure RAl, which consisted of two 2 nm GaAs rectangular quantum wells separated by 10 nm AlGaAs barriers containing 15% Al encapsulated in a 30% Al spacer. The simulation shows weak interaction between the two QWs due to the overlap of the two electronic wavefunctions, with the hole states not interacting with each other. Figure 1b shows the result of the calculation regarding PAl. The active region consists of a parabolic AlGaAs QW, where the aluminum content varies from 30% down to 9% and the QW has a thickness of 25 nm. The modeling shows the presence of a level inside of the parabola with a wavefunction wider than in the previous case. Figure 1c shows the gain region of RBi, consisting of three 6.8 nm GaAsBi QWs with 7 nm GaAs barriers.

Figure 1d shows the active area of PBi, consisting of three 10 nm GaAsBi QWs with 10 nm GaAs barriers enclosed in an AlGaAs parabolic barrier with an Al content that varies between 30 and 0%. In this gain region, the QWs are confined and the wavefunctions are localized in the wells corresponding to the hole wavefunctions.

The presented laser structures were grown using a solid-source “Veeco GenXplor R&D” (St. Paul, MN, USA) MBE system on Si-doped ($2\times 10^{18}$ cm^−3^) n-type double-side polished 350 µm-thick GaAs substrates (100). The temperature was recorded with a “kSpace Associates BandiT” band edge thermometry system working in transmission mode. A similar growth procedure was performed for the highly doped GaAs contact and AlGaAs cladding layers for all four LDs. A standard degassing procedure was performed at 650 °C for ~10 min to remove the native oxide. All LD structures followed a similar design with high (>$2\times 10^{18}$ cm^−3^) doping by Si and Be n+ and p+ GaAs layers grown to ensure an Ohmic contact. The growth rates varied from 300 to 580 nm/h. Further, Al_0.5_Ga_0.5_As cladding layers doped to $5\times 10^{17}$ cm^−3^ were grown with a 650 to 1200 nm/h growth rate. The growth rates for both material systems were reduced to a range of 300 to 450 nm/h for the active regions of the devices in order to achieve high quality and precision of growth. Moreover, contrary to AlGaAs, the growth of GaAsBi active regions must be carried out under specific growth conditions. A growth interruption was made before starting the growth of the bismuth-containing QW layer. The interruption was necessary to decrease the substrate temperature to 350–370 °C, reduce and stabilize the As flux to closely match that of Ga, and deposit a Bi wetting layer. We used 350 °C for the growth of RBi, while 370 °C was used for the growth of PBi. Bi predeposition was performed to avoid the delayed incorporation described by Ludewig et al. [26]. The growth of GaAsBi layers was performed in Bi-rich conditions; thus, Bi incorporation was limited solely by the substrate temperature. A major difference in structure design was implemented for the laser structure RBi, which was grown with a 1000 nm thick AlAs sacrificial layer. This layer is used in further processing, and enables removal of substrate and its transfer onto an integration platform. Detailed information on the parameters of the device active area is provided in Table 1.

The parabolic design of the barriers was created using an analog grading technique, with the parabolic shape of the conduction band achieved by gradually decreasing the Al content from 30% to 0% in the case of laser diode PBi and to 9% for structure PAl. This led to a relatively intricate growth protocol in which the PGB was segmented into steps of four monolayers each and a gradual decrease in Al content was implemented. The growth times were progressively extended due to the lowered growth rate resulting from the reduction in Al flux.

## 3. Processing and Characterization

After MBE growth, the laser structures were cleaned in acetone and isopropyl alcohol baths at 80 °C for 10 min each, followed by rinsing in deionized water and drying on a hotplate at 120 °C for 10 min. Next, the AZ1518 photoresist was spin-coated at 4000 RPM for 30 s and baked at 100 °C for 60 s. The pattern of the designed structure containing 4 to 5 µm bars was exposed through the mask under a 75 mW/cm^2^ dose of UV illumination. The developing process was carried out in H_2_O:AZ351B = 4:1 solution. The mesas were etched using a phosphorus acid solution of H_2_O:H_2_O_2_:H_3_PO_4_ = 10:1:1 for 3 min, stopping in the AlGaAs:Si cladding layer. The photoresist was removed by rinsing the sample in acetone, isopropyl alcohol, and deionized water, followed by O_2_ plasma cleaning for 5 min. To enable testing of the laser RBi and confirm its operational performance without carrying out the transfer, a different processing procedure was selected for a structure that was grown in preparation for transfer onto SiC platform. This particular diode structure was grown on a 1000 nm thick AlAs sacrificial layer for substrate removal. In order to test the properties of the laser prior to the substrate removal step, the processing procedure was modified compared to the other three diodes. The classical way of making the bottom contact is to deposit it on the substrate side; in this case, however, this was impossible due to the isolating AlAs layer between the substrate and the grown diode. Thus, an additional area was etched down to the n-type GaAs region and contacts of both n and p type were deposited from the sample side. For structure PBi, a part of the laser diodes were thinned. The final substrate thickness was 100 µm, which was achieved by both chemical (etching) and mechanical thinning. Metallization of the Ohmic contacts was accomplished by using an electron beam (VST Model TFDS-870, Petah-Tikva, Israel) to deposit Ti (15 nm)–Au (180 nm) on the p-type substrate and AuGe (140 nm)–Ni (25 nm)–Au (40 nm) on the n-type substrate (or n+ GaAs layer in the case of structure RBi). The laser bars were mechanically cleaved to form the ~500 µm Farby–Perot resonator. Electrical pumping of the laser diodes was achieved using two setups. A continuous-wave (CW) Agilent U8002A (Santa Clara, CA, USA) single-output DC power supply was used for the AlGaAs lasers, and output power injection current curves were recorded using a Thorlabs PDA50B-EC - Ge (Newton, NJ, USA) switchable gain detector. The spectra of the investigated AlGaAs-based devices were registered in CW mode using a Q8341 optical spectrum analyzer (Advantest, Tokyo, Japan ) with a wavelength resolution of 0.01 to 0.05 nm. All measurements were performed at room temperature (293 K). Prior to the experiment, all samples were mounted on gold-plated copper heat sinks using silver paste. A thermoelectric Peltier cooler was used for temperature stabilization. GaAsBi–based lasers were measured via pulsed electrical pumping using an AVTECH high voltage pulse generator (AVRZ-5W-B, Nepean, ON, Canada) operating at a 1 kHz repetition rate with a pulse duration of 50 ns. A digital handheld optical power meter (Thorlabs PM100D, Newton, NJ, USA)was used to register the output power. The lasing spectrum was dispersed through a 0.42 m monochromator VEB Carl Zeiss SPM-2 (Jena, DDR), and detected with a thermoelectrically cooled InGaAs photodetector IGA-030-TE2-H (Electro-Optical Systems Inc., Phoenixville, PA, USA). Current–voltage (*I-V*) characteristics under CW operation were measured using a B2901 precision source measurement unit (Keysight, Santa Rosa, CA, USA)with a voltage step of 10 mV. The maximum permissible DC current and forward voltage were limited to avoid damage to the quantum well region.

## 4. Results and Discussion

### 4.1. I-V Characteristics

**Figure 2:**
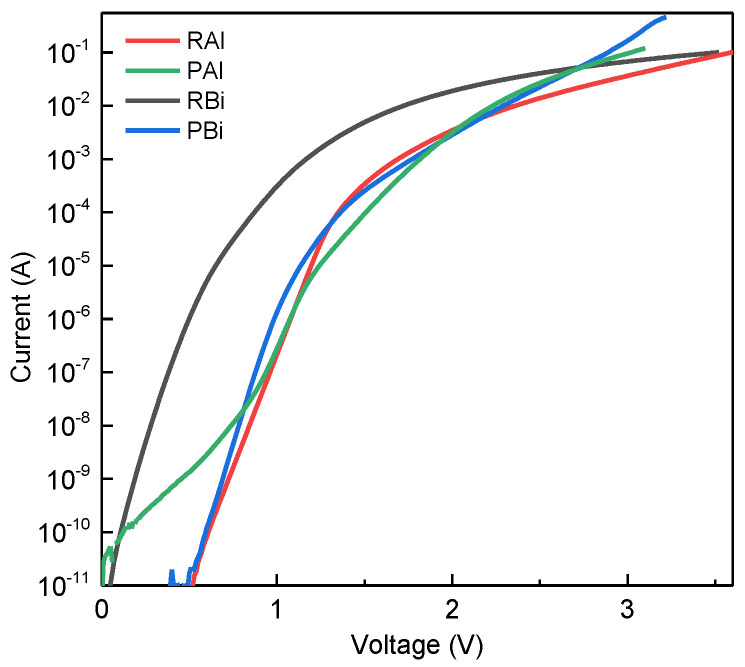
Current–voltage characteristics of the investigated laser diodes.

The *I-V* characteristics of the investigated laser diodes are typical for pn junction devices and can be expressed as $I=I_{0}\left(e^{\frac{q(U-IR_{s})}{\left(nkT\right)}}-1\right)$, where *I*_0_ is the saturation current of the diode, *q* is the elementary charge, *Rs* is the series resistance of the diode, *n* is the non-ideality factor of the diode, and *k* is Boltzmann’s constant. The non-ideality factor varies in the range from 2 to 2.5 in the laser diodes presented in Figure 2 for the current interval of 10^−7^ to 10^−5^ A. Usually, a factor close to 2 shows a dominant generation–recombination current component in a p-n junction [27]. The deviation from the exponential law at currents above 1 mA can be explained by the voltage drop on LD series resistance.

Here, laser diode PAl is distinguished by a higher non-ideality factor in the low-current range below 10^−8^ A, which is attributed to the leakage current component. This usually indicates slightly lower quality of the device/structure due to higher defectiveness, and is not necessarily related to the quantum well region of the LD. Our subsequent measurements of optical characteristics confirmed this, with no negative effect on LD performance observed above the threshold at this stage of characterization. However, the leakage current can contribute to reduced LD reliability during operation and shorter lifetime of the device [28,29]. A more detailed discussion of the electric measurements, including the *I-V* curve and the derivation of the non-ideality factor, can be found in [16].

### 4.2. Light Output Characteristics

**Figure 3:**
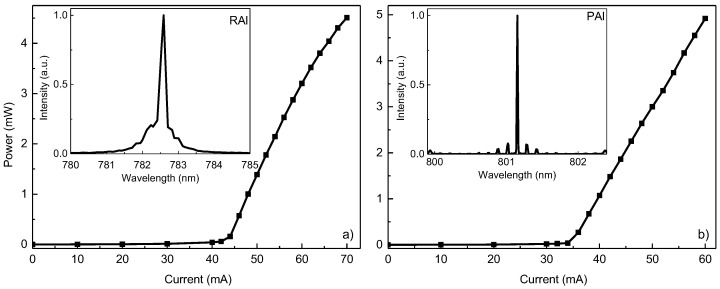
Room temperature measurement of output power versus injection current dependencies of the LDs. The insets show the emission spectra for (**a**) laser RAl and (**b**) laser PAl.

Figure 3 presents the injection current–output power characteristics measured for the two AlGaAs-based lasers, containing the standard rectangular design of the two QWs (Figure 3a) and a single parabolic QW design (Figure 3b). The lasing spectra of diodes RAl and PAl are shown in the insets of Figure 3a,b. The two lasers have similar emission lines at 801 and 780 nm, respectively. These values are good matches for the transition energy obtained by the nextnano++ modeling. The main lasing mode is mirrored by satellite Farby–Perot modes that are usual for as-cleaved laser diodes. The output power versus injection current characteristics reveal some inherent differences in the behavior of the two structures when a CW electrical current is applied. Even though the maximum output power measured for both diodes was similar, laser PAl (Figure 3b) had a higher output when comparing powers at the same injection current (60 mA), with 4.9 mW compared to 3.2 mW for laser RAl (Figure 3a). Furthermore, a decrease in threshold current (*I*_th_) is observed, from 43 mA for LD RAl to 35 mA for LD PAl. Finally, a gradual loss in the linearity of the dependence for laser RAl becomes noticeable at around 55 mA of injection current, showing power saturation. Such results are rather unusual, as the longer-wavelength laser demonstrates better parameters. We propose that PGBs in LD PAl are the reason for the enhanced device properties. Even though the nextnano++ simulation and *I-V* characteristics would suggest that the design, confinement, and quality of laser PAl are less optimal, the enhanced carrier capture efficiency via PGBs proposed by Pūkiene et al. has a more significant effect on the laser performance [20].

**Figure 4:**
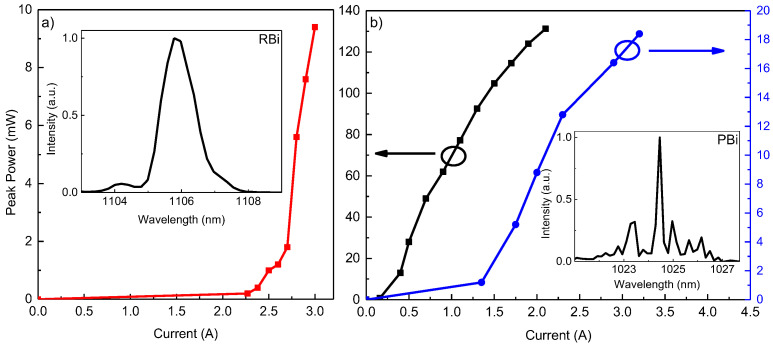
Room temperature measurement of output power versus injection current (pulsed 50 ns at 1 kHz) dependencies of the LDs. The insets show the emission spectra: (**a**) laser RBi and (**b**) laser PBi. The black curve represents the laser on the thinned substrate, while the blue curve represents the laser before substrate thinning.

CW operation of GaAsBi-based laser diodes could not be achieved due to problems related to defect formation promoted by the low-temperature growth and narrow window of As and Ga flux ratios discussed earlier in this paper. The peak output power versus injection current plots and lasing spectra obtained under pulsed excitation (50 ns at 1 kHz) are depicted in Figure 4, and are discussed separately. Lasing from the two diodes was recorded at a central wavelength of 1106 nm for LD RBi and 1024 nm for LD PBi. The redshifted lasing wavelength of laser RBi is attributed to the higher Bi content in the GaAsBi QWs, which is estimated to be at ~7% and ~5% for structures RBi and PBi, respectively. The trend of enhanced laser parameters employing PGBs is maintained for GaAsBi-based emitters. The maximum output power was doubled from ~10 mW to ~20 mW at 3 A of injection current, in addition to a twofold decrease in *I*_th_ (2.5 A and 1.3 A) for the sample with the improved barrier design. Moreover, a laser diode with a thinned substrate was fabricated (Figure 4b black curve) and a drastic improvement was noticed. The peak pulse output power was increased by almost an order of magnitude, to more than 130 mW, and a further decrease in *I*_th_ was noticed, achieving values as low as 250 mA. Such a significant effect of substrate thinning can be explained by two factors. First, the crystalline quality of the substrates is substantially worse than that of epitaxially grown layers. This means that the larger defect density results in loss of carriers in the substrate, leading to fewer of them reaching the active region. Second, the high defectiveness of bismides results in high resistance and significant local heating of the structure. The substrate thinning greatly enhances the temperature exhaust capabilities.

## 5. Conclusions

Application-focused QW LDs were fabricated in an attempt to improve the accuracy of pulse oximeters. LDs emitting in the range from 780–800 nm were produced using an AlGaAs material system. A maximum lasing output power of 4.9 mW was registered in the CW regime for the laser with PGBs emitting at 801 nm (Figure 3b), while the 782 nm laser with a classical rectangular design achieved 3.2 mW at the same injection (Figure 3a). GaAsBi alloy-based QW emitters exhibited lasing in the pulsed regime. A maximum peak output power of ~10 mW was recorded at a target wavelength of 1106 nm, where a classical rectangular design was used (Figure 4a). For a laser diode with three rectangular QWs embedded in PGBs, the recorded peak output power was 20 mW at 1025 nm (Figure 4b). These results confirm our hypothesis that improved carrier trapping efficiency can enhance LD performance. Both material systems showed improved output power and lasing threshold. The improvement of the AlGaAs samples was not as pronounced as for the GaAsBi ones, where both parameters were improved twofold. The reduced impact on the properties of AlGaAs-based LDs can be explained by the simulation results (Figure 1b) and the *I-V* curves (Figure 2), hinting at suboptimal quality and design of laser PAl. Additional steps were carried out to investigate the impact on substrate thinning for the optimized GaAsBi-based LD PBi. A decrease in the threshold current of over five times in magnitude and more than six times increased output power was recorded, with 250 mA of threshold current and over 130 mW of peak power produced by a GaAsBi-based LD in pulsed-mode operation.

## Figures and Tables

**Table 1:** Structural parameters of the intrinsic regions of grown devices.

Laser Diode	RAl	PAl	RBi	PBi
QW type	RQW	PGBs	RQW	RQW in PGBs
No of QW	2	1	3	3
QW composition	(Al, Ga)As		GaAsBi	
QW thickness, nm	2	25	6.8	10
Parabolic grading	-	Al_0.30_ ↔ Al_0.09_	-	Al_0.30_ ↔ Al_0.00_
Barrier composition	Al_0.15_Ga_0.85_As	-	GaAs	GaAs
Outer/inner barrier thickness, nm	5/10	-	-/7	-/10
Spacer composition	Al_0.30_Ga_0.70_As	Al_0.30_Ga_0.70_As	GaAs	-
Spacer thickness, nm	200	200	25	-

## Data Availability

The raw data supporting the conclusions of this article will be made available by the authors on request.

## References

[1] Sinex J.E. (1999). Pulse oximetry: Principles and limitations. Am. J. Emerg. Med..

[2] Yossef Hay O., Cohen M., Nitzan I., Kasirer Y., Shahroor-Karni S., Yitzhaky Y., Engelberg S., Nitzan M. (2018). Pulse oximetry with two infrared wavelengths without calibration in extracted arterial blood. Sensors.

[3] Francoeur S., Seong M.J., Mascarenhas A., Tixier S., Adamcyk M., Tiedje T. (2003). Band gap of GaAs_1-x_Bi_x_, 0<*x*<3.6%. Appl. Phys. Lett..

[4] Alberi K., Dubon O.D., Walukiewicz W., Yu K.M., Bertulis K., Krotkus A. (2007). Valence band anticrossing in GaAs_1-x_Bi_x_. Appl. Phys. Lett..

[5] Oe K., Okamoto H. (1998). New semiconductor alloy GaAs_1-x_Bi_x_ grown by metal organic vapor phase epitaxy. Jpn. J. Appl. Phys..

[6] Tominaga Y., Oe K., Yoshimoto M. (2010). Low temperature dependence of oscillation wavelength in GaAs_1-x_Bi_x_ Laser by photo-pumping. Appl. Phys. Express.

[7] Wu X., Pan W., Zhang Z., Li Y., Cao C., Liu J., Zhang L., Song Y., Ou H., Wang S. (2017). 1.142 *μ*m GaAsBi/GaAs quantum well lasers grown by molecular beam epitaxy. ACS Photonics.

[8] Ludewig P., Knaub N., Hossain N., Reinhard S., Nattermann L., Marko I.P., Jin S.R., Hild K., Chatterjee S., Stolz W. (2013). Electrical injection Ga(AsBi)/(AlGa)As single quantum well laser. Appl. Phys. Lett..

[9] Butkutė R., Geižutis A., Pačebutas V., Čechavičius B., Bukauskas V., Kundrotas R., Ludewig P., Volz K., Krotkus A. (2014). Multi-quantum well Ga(AsBi)/GaAs laser diodes with more than 6% of bismuth. Electron. Lett..

[10] Marko I.P., Ludewig P., Bushell Z.L., Jin S.R., Hild K., Batool Z., Reinhard S., Nattermann L., Stolz W., Volz K. (2014). Physical properties and optimization of GaBiAs/(Al)GaAs based near-infrared laser diodes grown by MOVPE with up to 4.4% Bi. J. Phys. D Appl. Phys..

[11] Fuyuki T., Yoshida K., Yoshioka R., Yoshimoto M. (2014). Electrically pumped room-temperature operation of GaAs_1-x_Bi_x_ laser diodes with low-temperature dependence of oscillation wavelength. Appl. Phys. Express.

[12] Armalytė S., Glemža J., Jonkus V., Pralgauskaitė S., Matukas J., Pūkienė S., Zelioli A., Dudutienė E., Naujokaitis A., Bičiūnas A. (2023). Low-frequency noise characteristics of (Al, Ga)As and Ga(As, Bi) quantum well structures for NIR laser diodes. Sensors.

[13] Luna E., Puustinen J., Hilska J., Guina M. (2024). Detection of Bi_Ga_ hetero-antisites at Ga(As,Bi)/(Al,Ga)As interfaces. J. Appl. Phys..

[14] Beyer A., Knaub N., Rosenow P., Jandieri K., Ludewig P., Bannow L., Koch S.W., Tonner R., Volz K. (2017). Local Bi ordering in MOVPE grown Ga(As,Bi) investigated by high resolution scanning transmission electron microscopy. Appl. Mater. Today.

[15] Gelczuk Ł., Kopaczek J., Rockett T.B.O., Richards R.D., Kudrawiec R. (2017). Deep-level defects in n-type GaAsBi alloys grown by molecular beam epitaxy at low temperature and their influence on optical properties. Sci. Rep..

[16] Glemža J., Špokas A., Zelioli A., Kamarauskas M., Bičiūnas A., Čechavičius B., Spigulis J., Chiu Y.J., Pralgauskaitė S., Matukas J. (2025). Quality evaluation of NIR laser diodes for medical application using low-frequency noise characterization. Infrared Phys. Technol..

[17] Puustinen J., Hilska J., Guina M. (2019). Analysis of GaAsBi growth regimes in high resolution with respect to As/Ga ratio using stationary MBE growth. J. Cryst. Growth.

[18] Richards R.D., Bastiman F., Hunter C.J., Mendes D.F., Mohmad A.R., Roberts J.S., David J.P.R. (2014). Molecular beam epitaxy growth of GaAsBi using As_2_ and As_4_. J. Cryst. Growth.

[19] Rockett T.B.O., Richards R.D., Gu Y., Harun F., Liu Y., Zhou Z., David J.P.R. (2017). Influence of growth conditions on the structural and opto-electronic quality of GaAsBi. J. Cryst. Growth.

[20] Pūkienė S., Karaliūnas M., Jasinskas A., Dudutienė E., Čechavičius B., Devenson J., Butkutė R., Udal A., Valušis G. (2019). Enhancement of photoluminescence of GaAsBi quantum wells by parabolic design of AlGaAs barriers. Nanotechnology.

[21] Andreani L.C., Pasquarello A., Bassani F. (1987). Hole subbands in strained GaAs - Ga_1-x_Al_x_As quantum wells: Exact solution of the effective-mass equation. Phys. Rev. B Condens. Matter.

[22] Mahtab M., Synowicki R., Bahrami-Yekta V., Bannow L.C., Koch S.W., Lewis R.B., Tiedje T. (2019). Complex dielectric function of GaAs_1-x_Bi_x_ as a function of Bi content. Phys. Rev. Mater..

[23] Ferhat M., Zaoui A. (2006). Structural and electronic properties of III-V bismuth compounds. Phys. Rev. B Condens. Matter Mater. Phys..

[24] Janotti A., Wei S.H., Zhang S.B. (2002). Theoretical study of the effects of isovalent coalloying of Bi and N in GaAs. Phys. Rev. B Condens. Matter.

[25] Karpus V., Norkus R., Butkutė R., Stanionytė S., Čechavičius B., Krotkus A. (2018). THz-excitation spectroscopy technique for band-offset determination. Opt. Express.

[26] Ludewig P., Knaub N., Stolz W., Volz K. (2013). MOVPE growth of Ga(AsBi)/GaAs multi quantum well structures. J. Cryst. Growth.

[27] Hu Z., Nomoto K., Song B., Zhu M., Qi M., Pan M., Gao X., Protasenko V., Jena D., Xing H.G. (2015). Near unity ideality factor and Shockley-Read-Hall lifetime in GaN-on-GaN *p-n* diodes with avalanche breakdown. Appl. Phys. Lett..

[28] Song Y., Lv Z., Bai J., Niu S., Wu Z., Qin L., Chen Y., Liang L., Lei Y., Jia P. (2022). Processes of the reliability and degradation mechanism of high-power semiconductor lasers. Crystals.

[29] Jiménez J. (2003). Laser diode reliability: Crystal defects and degradation modes. C. R. Phys..

